# Medical vs. Surgical Obesity Management: A Narrative Review of Efficacy, Safety, and Long-Term Outcomes

**DOI:** 10.7759/cureus.91677

**Published:** 2025-09-05

**Authors:** Joyce Akwe, Kimela Fongeh, Sarah Jung, Mary Ann Kirkconnell Hall, Viniya Patidar, Yoo Mee Shin

**Affiliations:** 1 Division of Hospital Medicine, Emory University School of Medicine, Atlanta, USA; 2 Department of Hospital Medicine, Atlanta Veterans Affairs Medical Center, Atlanta, USA; 3 College of Arts and Sciences, Emory University, Atlanta, USA; 4 Department of Medicine, Emory University School of Medicine, Atlanta, USA

**Keywords:** bariatric surgery, glp-1, glucagon-like peptide-1 receptor agonists, medical management of obesity, morbid obesity, obesity, overweight, overweight and obesity

## Abstract

Obesity is a chronic, relapsing, and multifactorial disease with rising prevalence and substantial health and economic burdens. Despite significant advances in both medical and surgical treatments, clinicians and patients still face a lack of clear, comparative guidance for tailoring therapy based on individual risk factors, disease severity, and comorbidities.

This review synthesizes current evidence on lifestyle and behavioral interventions, pharmacologic therapies (including glucagon-like peptide-1 [GLP-1] receptor agonists), and surgical approaches. Key clinical considerations in treatment selection include body mass index, eligibility for surgical or non-surgical interventions, patient preferences, tolerance for side effects, and the long-term impact on quality of life.

While lifestyle and behavioral modifications alone pose minimal risks, they are considerably less effective than when combined with pharmacologic or surgical/endoscopic treatments. Current pharmacotherapies, particularly GLP-1 receptor agonists and dual agonists such as tirzepatide, represent a paradigm shift in non-surgical weight management, though questions remain regarding their long-term efficacy and safety.

Bariatric surgery offers the most effective and durable weight loss outcomes, significantly improving obesity-related comorbidities and long-term survival. However, it carries higher upfront risks, potential complications, nutritional deficiencies, and increased costs.

Ultimately, integrated treatment models that combine lifestyle, pharmacologic, and/or surgical strategies are likely to offer the most effective and sustainable outcomes in managing obesity. Our review highlights the importance of an individualized, evidence-based, and multimodal strategy to effectively manage obesity and address its complexity. Continued research is essential to refine patient selection and improve integration of treatment options, ultimately supporting more personalized and effective obesity care.

## Introduction and background

Obesity is a chronic, relapsing, and multifactorial disease that is one of the most significant public health challenges of the 21st century. The World Health Organization (WHO) estimates that over 650 million adults worldwide would be classified as obese [1]. Obesity is defined as body mass index (BMI) ≥30 kg/m², and severe obesity as BMI ≥40 kg/m². In the United States, about 100 million people are obese, and about 22 million are severely obese [1]. From 1999 to 2020, the prevalence of obesity increased from 30.5% to 41.9%, while severe obesity increased from 4.2% to 9.2% [2].

Obesity is associated with numerous comorbidities, including type 2 diabetes mellitus (T2DM), hypertension, dyslipidemia, cardiovascular disease, obstructive sleep apnea, and certain cancers [3]. Obesity is estimated to contribute to approximately 2.8 million deaths per year globally and represents a significant economic burden due to healthcare costs and loss of productivity [1].

Lifestyle modifications through diet, exercise, and compassionate counselling have limited efficacy for individuals with severe obesity or obesity (BMI ≥35 kg/m²) with comorbidities [4]. The American Gastroenterology Association’s (AGA) Clinical Practice Guideline on Pharmacological Interventions for Adults with Obesity strongly recommends the use of pharmacotherapy in addition to lifestyle intervention in this group of adults [3].

Pharmacotherapy has been increasingly utilized to augment weight loss, with glucagon-like peptide-1 receptor agonists (GLP-1RAs), such as semaglutide and tirzepatide, demonstrating promising results in achieving clinically meaningful weight reduction [5,6]. AGA suggests the use of semaglutide 2.4 mg, liraglutide 3.0 mg, phentermine-topiramate extended release (ER), naltrexone-bupropion ER and phentermine, and diethylpropion (based on low certainty evidence), for long-term management of overweight and obesity [3].

However, like lifestyle management approaches, medical management often fails to achieve sustained weight loss in patients with severe obesity, highlighting the need for alternative or adjunctive approaches. Additionally, surgical technique advancements and long-term evidence support early consideration of bariatric surgery, including among patients with class I obesity (BMI 30-34.9 kg/m²) and significant metabolic disease [7].

Bariatric surgery has demonstrated superior outcomes compared to non-surgical interventions in terms of weight loss, lipid profile (total cholesterol, triglycerides, high-density lipoprotein [HDL]), blood pressure (systolic and diastolic), glycemic control (HbA1c), and overall cardiovascular risk across most clinical parameters [8]. Despite these advantages, bariatric surgery is associated with operative risks, nutritional deficiencies, and postoperative complications (as well as high initial cost), prompting ongoing research to optimize patient selection and improve long-term outcomes.

Comparing the efficacy, safety, and long-term outcomes of medical versus surgical interventions for obesity management is essential for developing a more comprehensive treatment framework. This review aims to critically evaluate the strengths and limitations of both approaches, providing insights into individualized obesity management.

## Review

In Spring 2025, we conducted a comprehensive narrative review by systematically searching PubMed, Google Scholar, Scopus, Web of Science, and EMBASE databases for relevant literature published between 2015 and 2025. We further hand-searched for highly salient older papers as well. Keywords included “obesity,” “medical management,” “pharmacotherapy,” “bariatric surgery,” and “surgical treatment.” Studies were selected based on relevance, quality, and direct comparison between medical and surgical approaches. Priority was given to randomized controlled trials, meta-analyses, and high-quality cohort studies. Only English-language articles were included.

Medical management of obesity

Medical management of obesity primarily involves lifestyle modification, pharmacotherapy, and behavioral therapy, often used in combination to enhance weight loss outcomes [9].

Lifestyle Modification

Lifestyle modification is the foundational approach to obesity treatment and is universally recommended as first-line therapy. It includes structured programs that integrate dietary changes, increased physical activity, and behavior change strategies. Clinical guidelines recommend a daily caloric deficit of 500-750 kcal, often achieved through individualized or group-based interventions [10]. Comprehensive lifestyle modification programs typically provide weekly individual or group treatment sessions designed to modify eating and activity habits [11].

Dietary interventions, such as the Mediterranean diet, low-carbohydrate diets, intermittent fasting, and plant-based diets, have demonstrated efficacy in promoting short- to mid-term weight loss [12]. However, maintaining long-term adherence to dietary changes remains a significant challenge due to environmental, psychological, and socioeconomic barriers [12].

Physical activity is essential not only for weight loss but also for preventing weight regain and improving cardiometabolic health. The American College of Sports Medicine recommends at least 150 minutes per week of moderate-intensity aerobic exercise for adults, increasing to 200-300 minutes for weight maintenance [13]. Despite these recommendations, physical inactivity remains prevalent, particularly among individuals with obesity due to physical limitations, lack of time, or psychological barriers.

Pharmacotherapy

Pharmacotherapy is typically recommended for individuals with a BMI ≥30 kg/m² or ≥27 kg/m² with obesity-related comorbidities, who have not achieved sufficient weight loss with lifestyle modifications alone [3]. The current FDA-approved medications for long-term obesity management are summarized in Table 1 and reviewed below; a number of other investigational medications are in development, such as amycretin, maridebart cafraglutide, mazdutide, orforglipron, retatrutide, and survodutide [14].

**Table 1:** Medications currently approved by the US Food and Drug Administration for the treatment of obesity AGA, American Gastroenterology Association.

Medication	Mechanism of action	Mean weight loss (percentage vs placebo)	Common side effects
Tirzepatide	Dual incretin effect	15-20% [15]	Thyroid risk including thyrotoxicosis and gastrointestinal side effects, metabolic dysfunction, refractory idiopathic intracranial hypertension, headaches, and visual disturbances [15-17]
Semaglutide	Enhances satiety and delays gastric emptying	15% [18]	Nausea, vomiting, and risk of pancreatitis [19]
Liraglutide	Enhances satiety and delays gastric emptying	5-8% [20]	Gastrointestinal upset and gall bladder diseases [21]
Bupropion/naltrexone	Appetite regulation	4-5% [22,23]	Nausea, insomnia, increased blood pressure, and seizures [23-25]
Phentermine/topiramate	Sympathomimetic and anticonvulsant; central nervous system appetite suppression	8-10% [26]	Mood changes, paresthesia, dry mouth [27,28]. Phentermine is also frequently prescribed as a single agent with a similar side effect profile.
Exenatide	Stimulates insulin secretion	3-5% [29]	Nausea, vomiting, diarrhea, abdominal pain, nasopharyngitis, and injection-site nodule [29]
Dulaglutide	Stimulates insulin secretion	4.7-5.4% [30]	Nasopharyngitis, upper respiratory infections, diarrhea [30]
Lixisenatide	Stimulates insulin secretion	2-3% [31]	Injection-site reactions, nausea, diarrhea [31]
Cagrilintide	Amylin analog; decreases appetite	6-10% [32]	Nausea, diarrhea, injection-site reactions [32]
Albiglutide	Enhances satiety and delays gastric emptying	1-2% [17,33]	Nausea, diarrhea, injection-site reactions, pancreatitis, upper respiratory tract infections [33]
Diethylpropion	Central nervous system stimulant and anorexiant	9.8% [4,34,35]	Dry mouth, insomnia, various cardiovascular and central nervous system effects [4,34,36]
Orlistat (note: not recommended by the AGA)	Lipase inhibitor that decreases absorption	3% [37]	Oily stool, fecal urgency, vitamin deficiency [17,37,38]

Incretin-based therapies, including glucose-dependent insulinotropic polypeptide/GLP-1 dual receptor agonist tirzepatide [15] and the GLP-1 RAs semaglutide 2.4 mg [18] and liraglutide 3 mg [20], are considered first-line medications for weight loss management [39]; recent evidence indicates that the human amylin analog drug cagrilintide is also effective [32], particularly in concert with semaglutide [40,41]. Significant questions remain about the durability of weight loss following cessation of these agents.

Liraglutide and semaglutide suppress appetite and delay gastric emptying. In addition to appetite suppression and delayed gastric emptying, GLP-1 RAs also exert multifaceted effects, including anti-inflammatory, cardioprotective, and potentially neuroprotective properties [39]. Semaglutide 2.4 mg weekly has shown weight loss up to 15% of initial body weight in clinical trials [19]. Additional clinical trials have demonstrated weight loss of over 20% of initial body weight, which further emphasizes the advancements in pharmacologic obesity management [17]. Recently, combination therapies with semaglutide and amylin analogs have shown an even greater efficacy, demonstrating weight loss up to 17-20% in early trials, though trials are ongoing [42]. Additionally, a recent umbrella review consisting of 235 randomized controlled trials found that semaglutide and similar pharmacotherapies contributed to a reduction in waist circumference, body mass index, lipid profiles, and blood pressure; these findings demonstrated the varied benefits of these medications in the management of obesity [38].

Tirzepatide is a dual GIP/GLP-1 RA that demonstrates greater efficacy, with 22.5% total body weight loss reported in the SURMOUNT-1 trial [5]. More recent data, however, suggest that the fat mass reduction achieved by tirzepatide and other incretin-based therapies may be accompanied by a loss of lean muscle mass as well [43].

Naltrexone-bupropion [22] is a second-line pharmacotherapy. It is not as effective as GLP-1 RAs and phentermine-topiramate [26], has a twice-daily dosing, and there is uncertainty about cardiovascular safety [23,24]. It is a combination drug that affects the reward system and appetite regulation. Modest weight loss has been observed (about 5-9%), but the cardiovascular safety of naltrexone-bupropion has not been established [25].

Phentermine/topiramate ER is a sympathomimetic and anticonvulsant combination associated with 8-10% weight loss [27,28]. It causes weight loss in the first year of use. It is a reasonable option for individuals without cardiovascular disease (or uncontrolled hypertension) who cannot tolerate or access GLP-1 RAs. It has dose-dependent cognitive side effects and is contraindicated in pregnancy. Phentermine/topiramate is indicated for adults with BMI ≥30 kg/m^2^ or with a BMI ≥27 kg/m^2^ with at least one weight-related comorbidity (e.g., hypertension, diabetes, dyslipidemia) [27,28].

Orlistat is a gastrointestinal lipase inhibitor that reduces fat absorption. It produces modest weight loss (about 3-4%) but is associated with gastrointestinal side effects [37]. AGA does not recommend orlistat in its guidelines [3].

Each form of pharmacotherapy has unique clinical considerations, as summarized in Table 2.

**Table 2:** Clinical considerations in the use of pharmacotherapy for obesity GLP-1, glucagon-like peptide 1.

Category	Details/Criteria
Indications	According to current guidelines from the Endocrine Society and American Gastroenterological Association [3,44]: BMI ≥30 kg/m² (obesity) OR BMI ≥27 kg/m² with at least one weight-related comorbidity, such as type 2 diabetes mellitus, hypertension, dyslipidemia, obstructive sleep apnea, cardiovascular disease
Ideal candidates for pharmacotherapy [11,19,45]	Have failed to achieve or sustain weight loss with lifestyle interventions alone.
	Are motivated and capable of long-term adherence to medication and lifestyle changes.
	Do not have contraindications to specific medications (e.g., pregnancy, certain psychiatric conditions).
	Are at risk of complications from excess adiposity (e.g., insulin resistance, non-alcoholic fatty liver disease).
	Note: Shared decision-making is essential, considering efficacy, side effect profile, cost, comorbidities, and patient preference.
Benefits	Cardiometabolic improvement: HbA1c reduction, blood pressure lowering, improved lipids
	Reduced progression from prediabetes to type 2 diabetes mellitus
	Improved quality of life and physical functioning
	Cardiovascular risk reduction: The SELECT trial (semaglutide 2.4 mg) showed a 20% reduction in major adverse cardiac events in patients with obesity and established cardiovascular disease [46-48]
	Preservation of lean muscle mass: Combining newer pharmacologic agents with resistance training and adequate protein intake may help reduce potential muscle loss [43,48]
	Novel GLP-1RA combinations and triple agonists have shown potential benefits in metabolic comorbidities such as steatotic liver disease [42]
	Comprehensive health improvements: Favorable changes in cardiovascular and metabolic measures, including reductions in blood pressure, improved lipid profiles and glycemic control [38]
	GLP-1-based therapies have shown significant cardiovascular risk reduction, including lower rates of major adverse cardiovascular events, and emerging evidence suggests potential renoprotective effects as well [39]
	In patients with obesity and established heart failure with preserved ejection fraction, semaglutide significantly reduced cardiovascular mortality and heart failure hospitalization in a prespecified subgroup analysis of the SELECT trial [48]
Side effects and safety [3,5]	Gastrointestinal symptoms are the most common across GLP-1 agents
	Neuropsychiatric effects: Bupropion/naltrexone carries a warning for suicidal ideation
	Renal function and gallbladder disease: Monitor with GLP-1s
	Teratogenicity: Avoid phentermine/topiramate in women of childbearing age not using contraception
	Rare but serious: Pancreatitis (GLP-1s), seizures (bupropion), thyroid C-cell tumors (GLP-1s; mostly in rodents)
	Recent concerns include gallbladder disease, possible thyroid abnormalities, and rare reports of accelerated thyrotoxicosis [39]

Although pharmacotherapy represents a major advancement in obesity management, challenges remain. These include variability in response, side effects, cost, and insurance coverage barriers, and potential weight regain after discontinuation of the medication. These concerns are especially relevant in patients with heart failure, since unintentional lean mass loss in patients may worsen outcomes. Resistance training and close nutritional monitoring are advised in this population [48].

Behavioral Therapy

Behavioral interventions are a critical adjunct to both lifestyle and pharmacologic approaches. These programs focus on identifying maladaptive eating behaviors, enhancing self-monitoring skills, goal setting, and problem-solving strategies. Behavioral therapy delivered via intensive individual counseling, group sessions, or digital platforms has been shown to improve adherence and promote weight loss of up to 5-10% [11]. High-intensity face-to-face cognitive-behavioral therapy (CBT) shows better short-term outcomes, particularly for binge eating disorder and bulimia nervosa, which are often associated with obesity. However, evidence is limited for group CBT, low-intensity CBT, and long-term effects [49].

Recent studies highlight the importance of long-term support and follow-up in maintaining behavioral changes. Digital health tools, mobile apps, and telehealth coaching (e.g., Noom, Omada) are gaining traction for enhancing patient engagement and reducing barriers to access [50]. In light of newly emerging evidence, behavioral changes should include resistance training to minimize the potential loss in muscle mass sometimes seen with these pharmacologic agents [43].

In summary, while medical management of obesity continues to evolve with promising pharmaceutical and behavioral tools, its overall effectiveness is modest when compared to surgical interventions, especially in individuals with severe or refractory obesity. Medical therapy may, however, serve as an essential first-line treatment or a complement to surgical approaches depending on individual patient needs, comorbidities, and preferences.

Surgical and endoscopic management of obesity

Bariatric surgery remains the most effective long-term treatment for weight loss and metabolic improvement [51,52]. While sleeve gastrectomy (SG) and Roux-en-Y gastric bypass (RYGB) are still the most frequently performed procedures [53], recent trends reflect a shift toward less invasive, technically simpler surgeries, often used alongside pharmacotherapy to minimize complication risks.

Adjustable gastric banding (AGB) has fallen out of favor, now accounting for less than 2% of bariatric procedures in the US, due to limited long-term efficacy, high complication rates, and the need for frequent reoperations [54,55]. Although the traditional biliopancreatic diversion with duodenal switch (BPD/DS) is highly effective for weight loss and remission of metabolic disease [56-58], its use has declined because of significant concerns over nutritional deficiencies.

In response, newer surgical techniques have been developed to maintain metabolic benefits while reducing surgical complexity. The single anastomosis duodeno-ileal bypass with sleeve gastrectomy (SADI-S), a simplified version of the duodenal switch, has been endorsed by both the American Society for Metabolic and Bariatric Surgery (ASMBS) and the International Federation for the Surgery of Obesity and Metabolic Disorders (IFSO). It offers weight loss and metabolic outcomes comparable to BPD/DS, with potentially fewer nutritional risks [59-61]. Similarly, the one-anastomosis gastric bypass (OAGB), also recognized by ASMBS and IFSO, has demonstrated outcomes equivalent to RYGB in the YOMEGA randomized trial, while offering shorter operative times and greater technical simplicity [62].

Additional “sleeve-plus” procedures, such as the single anastomosis sleeve ileal (SASI) and sleeve jejunal (SASJ) bypass, combine SG with a partial intestinal bypass to harness incretin-driven metabolic effects while preserving some absorptive function. Early data suggest these procedures can yield over 70% excess weight loss at 12 months and high rates of diabetes remission, with complication rates similar to more established operations [63,64].

Endoscopic techniques are also expanding treatment options. Endoscopic sleeve gastroplasty (ESG), which mimics SG without gastric resection, has shown sustained weight loss in patients with class I-II obesity [65,66], and its effectiveness appears to be enhanced when combined with GLP-1 RAs [67]. For patients experiencing weight regain after RYGB, transoral outlet reduction endoscopy can restore satiety and alleviate dumping syndrome by reducing the gastrojejunal outlet size, with outcomes further improved when paired with pharmacotherapy [68,69]. These evolving strategies offer less invasive, metabolically meaningful alternatives, which are especially relevant in the context of modern pharmacologic therapies.

Comparison of bariatric versus non-surgical management of obesity

Comparative Effectiveness: Patient Outcomes

In a meta-analysis performed by Pipek et al. [8], surgical options were favored over non-surgical options. Overall, surgical options provide the best outcomes (Table 3). The amount of weight loss is higher, and remission of type 2 diabetes, hypertension, and metabolic syndrome is favorable [8]. Although peri- and post-surgical complications are present in surgical options, it is important to think about the long-term health effects of obesity that is not treated adequately. If patients are not able to achieve improvement in glycemic control, it can cause downstream health effects such as an increase in cardiovascular and cerebral comorbidities. 

**Table 3:** Comparative analysis of bariatric vs non-surgical management of obesity GLP-1 RA, glucagon-like peptide 1 receptor agonist; LDL, low-density lipoprotein; HDL, high-density lipoprotein.

Variable	Bariatric surgery	Non-surgical treatment (includes lifestyle interventions ± medications)
Average weight loss	Better weight outcomes with loss of 26 kg as compared to non-surgical options [70]	Lifestyle intervention results in less weight loss, and average amount of weight loss varied with different medications [9]
	Total body weight loss of ~27% [51]	GLP-1 RAs and dual agonists: 15-20% weight loss in 2 years [71]
	Roux-en-Y is the better option as compared to non-surgical options [72]	
	Weight loss is more sustainable than with non-surgical options	
	Reduction in the overall incidence of obesity-related cancer and cancer-related mortality [73]	
Impact on co-morbid conditions (type 2 diabetes, hypertension, metabolic syndrome)	Better glycemic control with a difference of HbA1c of 1.4% at 7 years [74]	At 5 years, there were no patients who were able to get off medications for glycemic control (including insulin and oral agents) with lifestyle modification. In addition, improvements in blood pressure and cholesterol profile are not statistically significant compared to bariatric surgery [75]
	Improvement in systolic and diastolic blood pressure [8]	
	Decrease in LDL and triglycerides and increase in HDL [8]	
	Improvement in metabolic syndrome and cardiovascular events [76]	
Quality of life (QoL) post-intervention	Improvements in overall QoL with better mobility, self-care, decrease in pain although mental health scores may return to baseline post-surgery in patients who have bariatric surgery [77,78]	Patients have improvement in overall QoL due to better mobility, self-care, usual activities, and pain and also decrease in anxiety and depression. Patients who undergo lifestyle modification maintain the improvements in mental health better than those who had had surgery [78,79]
Risks	Peri- and postoperative complications (including reoperations, hernia, pneumonia, iron deficiency anemia, cholecystitis) can occur but complications are decreasing [70,80]	Lower short-term risk profile
		Uncertain safety profile of new medications including GLP-1 RAs [78]
		Side effects include gastrointestinal upset (nausea, vomiting, diarrhea), pancreatitis. In an animal study, there was a higher incidence of thyroid C-cell carcinoma and, therefore, liraglutide is contraindicated in patients with a history of medullary thyroid cancer for multiple endocrine neoplasia (MEN) type 2 (personal or family history) [81]

Comparative Cost-Effectiveness

When deciding between surgical options vs non-surgical options to treat obesity, it is important to consider the economic implications of both treatments. With surgical options, there is an upfront cost, but downstream health effects must also be considered, including complications from type 2 diabetes, cardiovascular disease, and cerebral vascular incidents that might occur if a patient does not seek treatment for obesity. In a study by Cohen et al. [82], investigators evaluated the cost difference for patients having early versus delayed bariatric surgery; they found that delaying bariatric surgery is overall more expensive than receiving prompt surgery. Hsu and Farrell further support this finding, reporting that reducing cardiovascular events and diabetes-related complications reduces downstream healthcare costs [83]. Caution is warranted, however: a study by Docimo et al. [84] revealed that the cost for certain GLP-1 medications would exceed the cost of surgical options within a year in the United States.

Summary

Selection between surgical and non-surgical interventions should be guided by established clinical guidelines, while also accounting for individual patient preferences, risk profiles, comorbid conditions, age, and economic considerations (Table 4).

**Table 4:** Patient selection criteria for surgery vs non-surgical treatment BMI, body mass index; DM, diabetes mellitus; OSA, obstructive sleep apnea.

	Bariatric surgery	Non-surgical treatment (includes lifestyle interventions ± medications)
BMI requirement	BMI ≥40 kg/m^2^ or BMI ≥35 kg/m^2^ with weight-related complication and when non-surgical interventions have failed	Patients with BMI ≥30 kg/m^2^ or BMI ≥27 kg/m^2^ and weight-related complication; BMI ≥28 kg/m^2^ and additional risk factors [85]
	For BMI ≥50 kg/m^2^, bariatric surgery should be the treatment regardless of whether non-surgical options have been pursued [85]	
Obesity-related conditions	Recommended for patients with severe obesity-related conditions including type 2 DM, heart disease, and OSA, which may improve post-surgery [85]	Recommended for those with mild to moderate obesity-related conditions
Cost	Higher upfront cost but more cost-effective in the long term [86]	Lower initial cost but with long-term expenses with medications [84]
Age	Consider for all adults who meet the criteria. Special consideration for patients under the age of 18 with morbid obesity who have gone through rigorous evaluation with a pediatrician and with parental involvement. Also, special consideration for patients above the age of 60 years but there is a higher risk of peri-operative complications [87]. It is important to consider long-term effects of bariatric surgery performed in children, including vitamin deficiencies from absorption complications and surgical complications [81].	No age restriction for lifestyle modification. Special consideration for medications for children 18 years old and younger.

Overall, obesity pharmacotherapy and bariatric surgery are underused resources when it comes to the management of obesity [81]. The primary considerations are BMI and whether patients meet the criteria for surgical or non-surgical treatment options [85]. Initially, most providers start with lifestyle modifications with or without medications. Special considerations need to be taken into account when thinking about starting a medication for obesity treatment. Patients will need to think about the side-effect profile, particularly those medications that have gastrointestinal effects that may not be well tolerated [66,81]. Once a patient has failed conservative management, they may be referred for bariatric surgery, especially if they have an obesity-related condition such as type 2 diabetes. Patients do require medical clearance before undergoing bariatric surgery, but surgery is generally well tolerated with few peri- and post-surgical complications [88,89]. 

As the global obesity epidemic intensifies, the need for effective, individualized, and sustainable management strategies has never been more critical. This narrative review underscores the distinct yet complementary roles of medical and surgical interventions in the treatment of obesity.

While pharmacologic therapies, particularly the emerging GLP-1 RAs and dual agonists like tirzepatide, represent a paradigm shift in non-surgical weight management, concerns have also emerged about their impact on lean muscle mass, which may limit their long-term utility if not addressed [90]. Additionally, newly developed combination therapies and triple agonists provide new avenues for more effective and comprehensive obesity management strategies [28]. For example, pharmacotherapies can provide cardiovascular and metabolic benefits as a component of obesity management [37]. Emerging evidence supports the earlier initiation of GLP-1-based therapies in individuals with metabolic syndrome or prediabetes, potentially delaying progression to overt disease [19]. Recent data also support the cardiovascular and functional benefits of GLP-1-based therapies in patients with heart failure with preserved ejection fraction, reinforcing their role in the comprehensive management of obesity-related heart failure [46-48].

In contrast, bariatric surgery continues to demonstrate superior efficacy in inducing durable weight loss, improving obesity-related comorbidities, and enhancing long-term survival, albeit with higher upfront risks and the potential for complications and nutritional deficiencies. Integrated treatment models that combine medical and surgical approaches have demonstrated superior outcomes in obesity management. While bariatric surgery is highly effective, maintaining long-term weight loss can be challenging. A multidisciplinary approach, incorporating nutritionists, exercise specialists, and psychologists, may prove to be more effective in optimizing patient outcomes. Further research is needed to explore the best ways to integrate these components into bariatric care.

## Conclusions

Optimal obesity care demands a patient-centered, multidisciplinary approach that integrates emerging pharmacotherapies with established surgical interventions. Future research should aim to identify predictors of treatment response, refine patient selection criteria, improve access to care, and develop integrated models that harness the complementary strengths of medical and surgical strategies. Long-term studies are also essential to evaluate the durability of health benefits and to monitor potential adverse effects associated with prolonged pharmacotherapy. Ultimately, progress in obesity management depends on a sustained commitment to evidence-based, equitable, and individualized care for this complex, chronic disease.
